# Publication Trends of Research on Sepsis and Host Immune Response during 1999-2019: A 20-year Bibliometric Analysis

**DOI:** 10.7150/ijbs.37496

**Published:** 2020-01-01

**Authors:** Ren-qi Yao, Chao Ren, Jun-nan Wang, Guo-sheng Wu, Xiao-mei Zhu, Zhao-fan Xia, Yong-ming Yao

**Affiliations:** 1 Trauma Research Center, Fourth Medical Center of the Chinese PLA General Hospital, Beijing 100048, People's Republic of China.; 2 Department of Burn Surgery, Changhai Hospital, the Naval Medical University, Shanghai 200433, People's Republic of China.; 3 Basic Medical College, the Naval Medical University, Shanghai 200433, People's Republic of China.

**Keywords:** Bibliometric analysis, Publication, Sepsis, Immune cell, Immunosuppression.

## Abstract

**Introduction:** Sepsis is an intractable disorder, which is associated with high risk of organ dysfunction and even death, while its pathogenesis remains largely unclear. Our study aims to study the research trend on sepsis and host immune response, and compare the contribution of publications from different countries, institutions, journals and authors.

**Materials and Methods:** We extracted all relevant publications with regard to sepsis and immune response during 1999-2019 from Web of Science. GraphPad Prism 6, and VOSviewer software were used to collect and analyze the publication trend in related field.

**Results:** We identified a total of 1225 publications with citation frequency of 40511 times up to March 30, 2019. The United States accounted for the largest number of publications (36.3%), 51.9% of total citations as well as the highest H-index (72). The sum of publications from China ranked the second, while the overall citations (1935) and H-index (22) ranked the eighth and the seventh, respectively. Journal of *Shock* had published most papers related to the topic on sepsis and immune response. Ayala A SA, has published the most papers in this field (31), while Hotchkiss RS presented with the most citation frequency (3532). The keyword “regulatory T cell” appeared most recently with an average appearing years of 2014.0. The “immunosuppression related research” seemed to be the hotspot in relevant scope.

**Conclusions:** The United States made the most outstanding contribution within this important field. There is a mismatch between the quantity and quality of publications from China. Latest progress can be tracked in journal of *Shock*. Immunosuppression related researches may be hotspots in the near future.

## Introduction

Sepsis remains one of the most common causes of death among patients admitted to intensive care units (ICU) 1, 2. In accordance with the third international consensus definition for sepsis and septic shock (Sepsis-3.0), sepsis is known as a life-threatening organ dysfunction, which is mainly caused by dysregulated host response to infection 3. Although enormous progress has been achieved in the early recognition, prevention, and treatment for septic cases, both the incidence and mortality of sepsis are still very high 4, 5. As decades of attempts by using anti-inflammatory measures to limit the devastating tissue damage due to excessive inflammation have been failed, no efficient therapies have been characterized at present 6.

It is consistently accepted that sepsis initiates a complex immune response with the concomitant occurrence of both pro-inflammatory and anti-inflammatory responses but presenting with abnormal homeostasis 7. Indeed, most septic patients initially experience a short-term hyperinflammation but subsequently suffer from a prolonged immunosuppression status which is significantly associated with late-stage mortality 8-10. Intrinsically, immunosuppression is demonstrated to attribute to dysfunction of various immune cells. For example, increased apoptosis of T cells, B cells, and dendritic cells (DC) has been noted and identified as a major cause for poor prognosis of patients with septic complications as a result of marked reduction of these cells in circulation 11-14. Besides, plenty of inhibitory immune receptors, including programmed death-1 (PD-1), cytotoxic T lymphocyte antigen-4 (CTLA-4), and B and T lymphocyte attenuator (BTLA) are manifested with marked up-regulation in the development of sepsis, which result in dysfunction of multiple immune cells, thereby hindering immune responses from eliminating pathogens' invasion 15-17. Thus, therapeutic methods targeting immunosuppression as well as reversal dysfunction of immune cells have drawn extensive attention worldwide.

Bibliometric analysis is an optimal choice for providing detailed trend of research activity in a certain field over time 18. By applying literature system and literature metrology characteristics as research objects, bibliometrics are employed to analyze publications, such as book or journal articles quantitatively and qualitatively. Other than characterizing and predicting development in a specific research field, this type of analysis can compare the contribution of disparate countries, institutions, journals, and scholars 19. Particularly, bibliometric analysis has played an important role in the formulating policy and clinical guideline of various diseases, which makes it become increasingly popular nowadays 20-23.

The present study aims at providing a comprehensive analysis of the current status of sepsis and immune response research based on Web of Science (WOS). We applied methodology of bibliometric analysis in order to uncover the research trend of certain field and predict its possible hotspot in the future.

## Materials and methods

### Data sources and search strategies

It has been consistently accepted that the Science Citation Index-Expanded (SCI-E) of Thomson Reuters' Web of Science is the most appropriate database for performing bibliometric analysis. We conducted a comprehensive online search from 1999 to 2018 by applying Web of Science database with document types restricted to original articles and reviews. All data were obtained through public database and had nothing to do with any human subject. Given that, ethical consent was not applicable.

**Figure 1:**
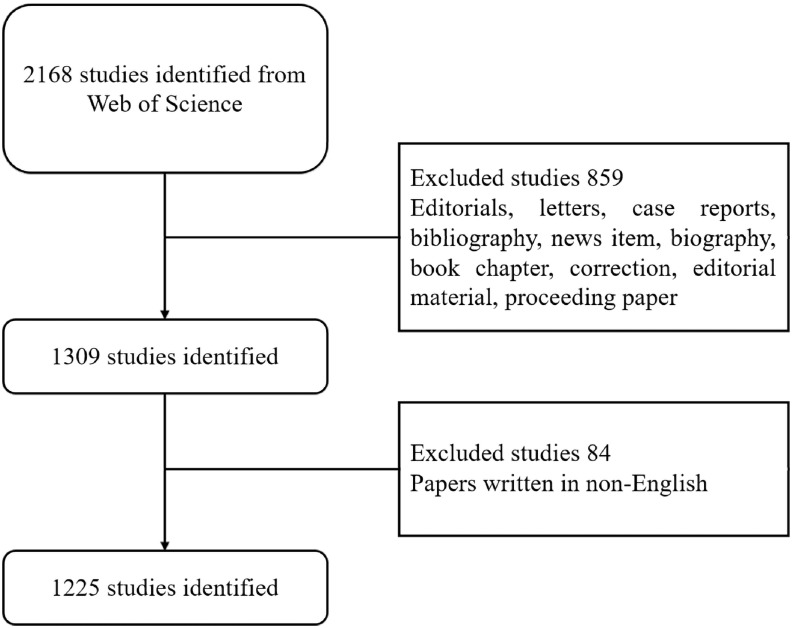
Flow diagram of the inclusion process. The detailed process of screening and enrollment.

All searches were conducted in a single day March 30, 2019 in order to avoid bias introduced by rapidly database renewal. The search strategies were presented as follows: TI = (sepsis OR (severe sepsis) OR (septic shock) OR (endotoxemia) OR SIRS OR (systematic inflammatory response syndrome)) AND TI = (macrophage OR neutrophil OR (NK cell) OR (natural killer cell) OR (dendritic cell) OR DC OR (innate lymphoid cells) OR ILCs OR (T cell) OR (T lymphocyte) OR (B cell) OR (B lymphocyte) OR (regulatory T cell) OR (Treg) OR (monocyte) OR immunosuppression OR (immune dysfunction) OR (immune response)) AND Language = English. Additionally, articles and reviews with normal peer-review were potentially eligible, while others were excluded accordingly. Detailed process of the enrollment and screening was shown in **Fig. 1**.

### Data collection

Two reviewers (RQY and CR) independently extracted data from all included publications, including titles, keywords, publication dates, countries and regions, authors, institutions, publishing journals, sum of citations, H-index, and so on. The data came from Web of Science were inputted into Microsoft Excel 2016 (Redmond, Washington, USA), GraphPad Prism 6 (GraphPad Prism Software Inc., San Diego, CA), VOSviewer (Leiden University, Leiden, the Netherlands), and subsequently analyzed quantitatively yet qualitatively.

### Bibliometric analysis

Web of Science was applied to describe the characteristics of all incorporated publications. Relative research interest (RRI) was defined as the number of publications related to a specific research field divided by publications across all fields per year 24. Impact factor (IF) was indicated by inquiring the latest version of Journal Citation Reports (JCR). It has been widely accepted that H-index serves to reflect the scientific research impact of a scholar or a country. The index of H means that a scholar/country has published H papers and each of which has been cited in other publications at least H times 25.

Microsoft Excel 2016 was applied to generate prediction model: f(x) = ax^3^+bx^2^+cx+d, in which we analyzed the time trend of the publications as well as the future change tendency based on the cumulative number of publications. In this formula, x stood for the year, and f(x) represented the cumulative volume of publications by the year.

VOSviewer is an optimal approach for analyzing the correlation of highly cited references with productive authors. In addition, it is commonly used to map and visualize the network of keywords that are related to sepsis and research on immune response 26. Furthermore, VOSviewer can classify keywords into disparate clusters in accordance with co-occurrence analysis, and simultaneously color them by time course. The definition of average appearing year (AAY) was applied to quantify the relative novelty of a keyword.

## Results

### Contribution of countries to global publications

**Figure 2:**
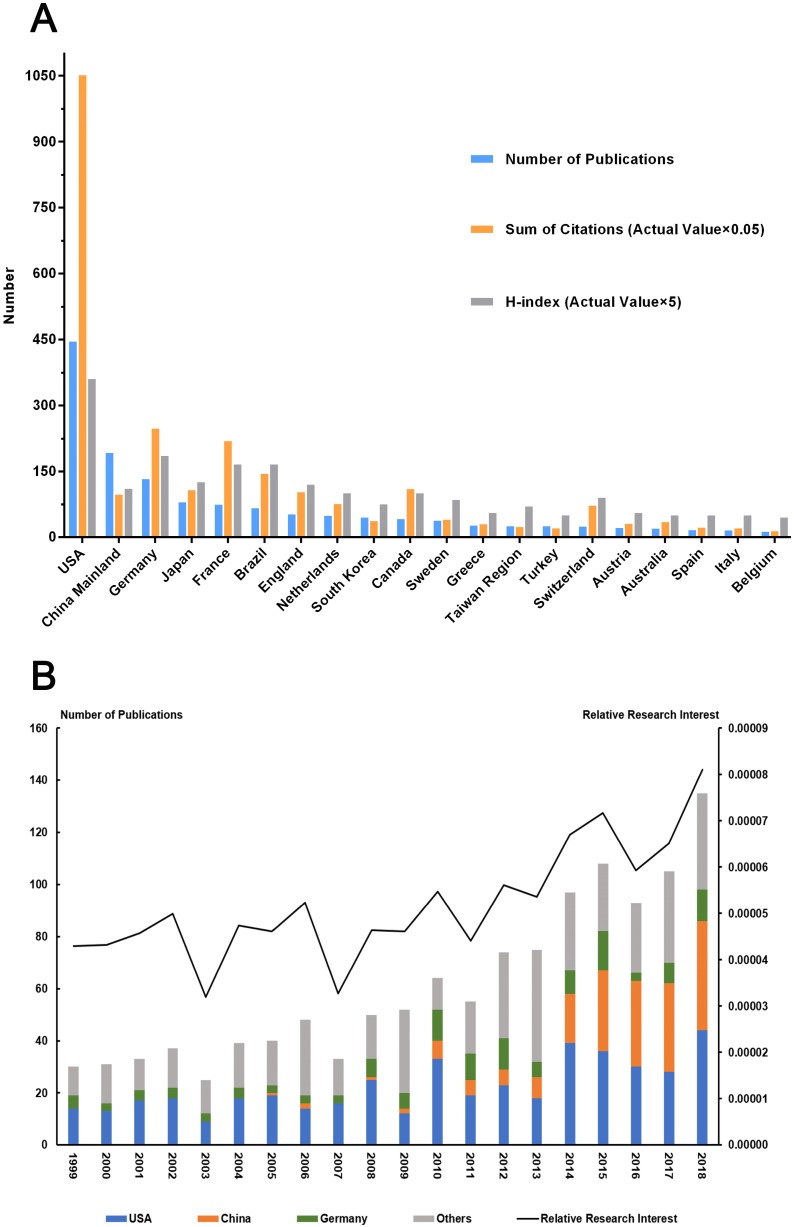
** Contributions of different countries/regions to the research field concerning sepsis and immune response.** (A) The number of publications, citation frequency (×0.05), and H-index (×5) in the top 20 countries or regions; (B) The number of publications from worldwide and the top 3 countries per year, and the time course of relative research interest of sepsis and immune response.

A total of 1225 articles from 1999 to 2018 met our inclusion criteria. The United States ranked the first in the number of publications (445, 36.3%), followed by China (192, 15.7%) and Germany (132, 10.8%). By calculating the number of papers per year, we found that publications were the most within the year of 2018 (136, 11.1%) (**Fig. 2A**). When the amount of all-field publications was taken into consideration, the global attention towards this certain field which measured by the value of RRI fluctuated around 0.005% before 2010, while subsequently went up and reached 0.008% at 2018 (**Fig. 2B**). Not until 2005 did Chinese researches initially publish papers in this field. However, the proportion of Chinese publications in this field has been rising rapidly for the past 8 years. Notably, China (33, 35.5%) exceeded the United States (30, 32.3%) in the number of publications for the first time in 2016.

### Citations and H-index analysis

By retrieving the citation report from the Web of Science database, all articles related to sepsis and immune responses were cited for 40511 times since 1999 (36049 times without self-citations). Average citing frequency was 33.07 times per paper. The United States accounted for 51.9% of the total citations, which were 21019 (19938 times without self-citations) with an H-index of 72. The number of citations of publications from Germany was 4945 (4865 times without self-citations) with an H-index of 37, which both ranked the second among all involved countries. Although the overall number of publications from China in this field ranked the second to the United States, the citation frequency was only 1935 times with an H-index of 22, which ranked the eighth and the seventh, respectively (**Fig. 2A**).

### Growth trends of publications

**Figure 3:**
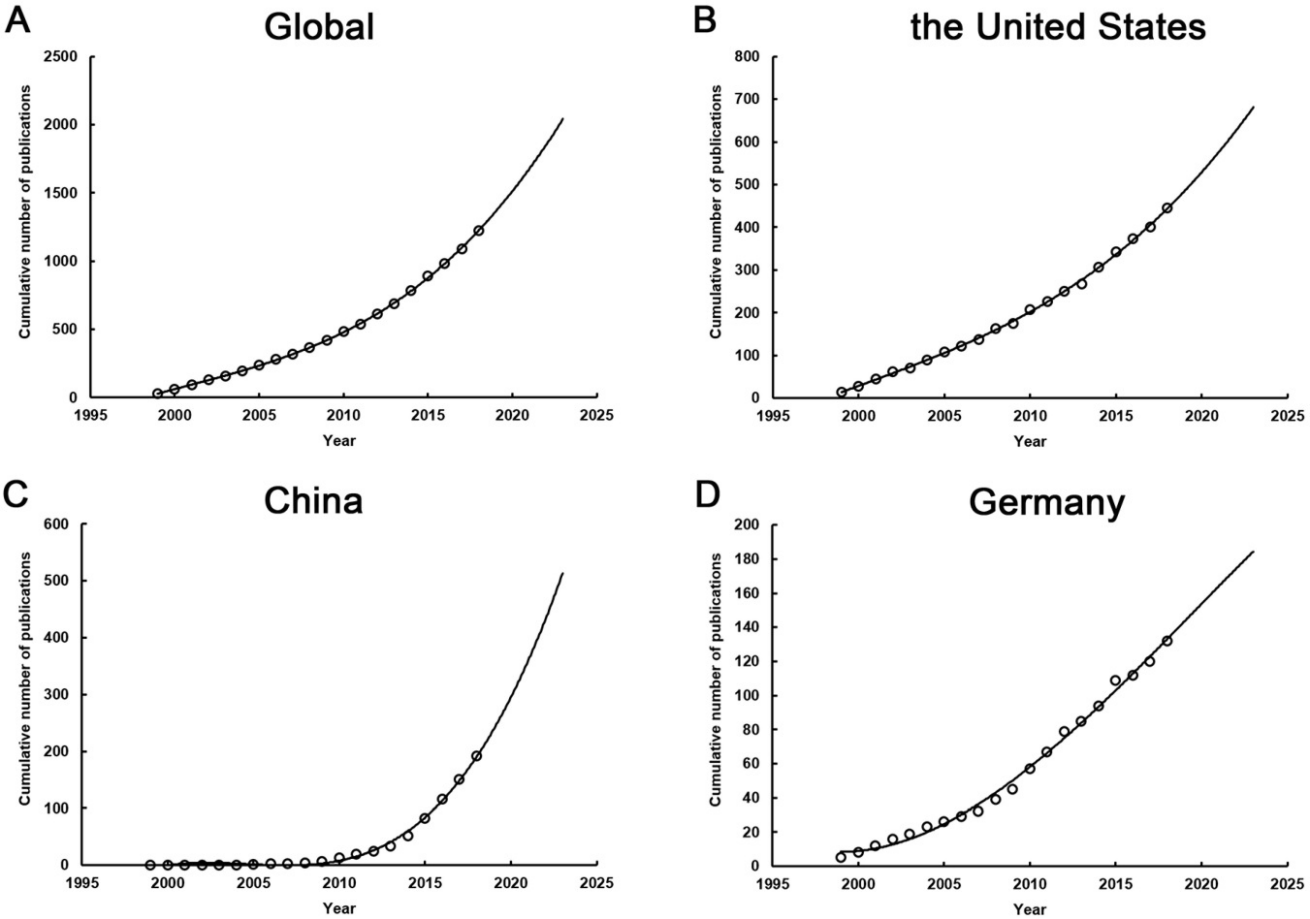
The model fitting curves of growth trends of publications associated with sepsis and immune response. (A) Global; (B) the United States; (C) China; (D) Germany.

Based on the model fitting curves of publication growth, the cumulative publication number of global and different countries was shown in **Fig. 3**. By the year of 2021, there were estimated 1675 papers for the entire world, 576 for the United States, 359 for China, and 164 for Germany. We found that the growth of publications for the entire world was on a slow curve, which was also applicable for several major countries, such as the United States and the Germany (**Fig. 3B and 3D**). The number of papers published by those countries per year remained unchanged in recent years, while the China showed an obviously faster growth curve in the form of three polynomial compared to other countries (**Fig. 3C**).

### Journals publishing researches on sepsis and immune response

**Figure 4:**
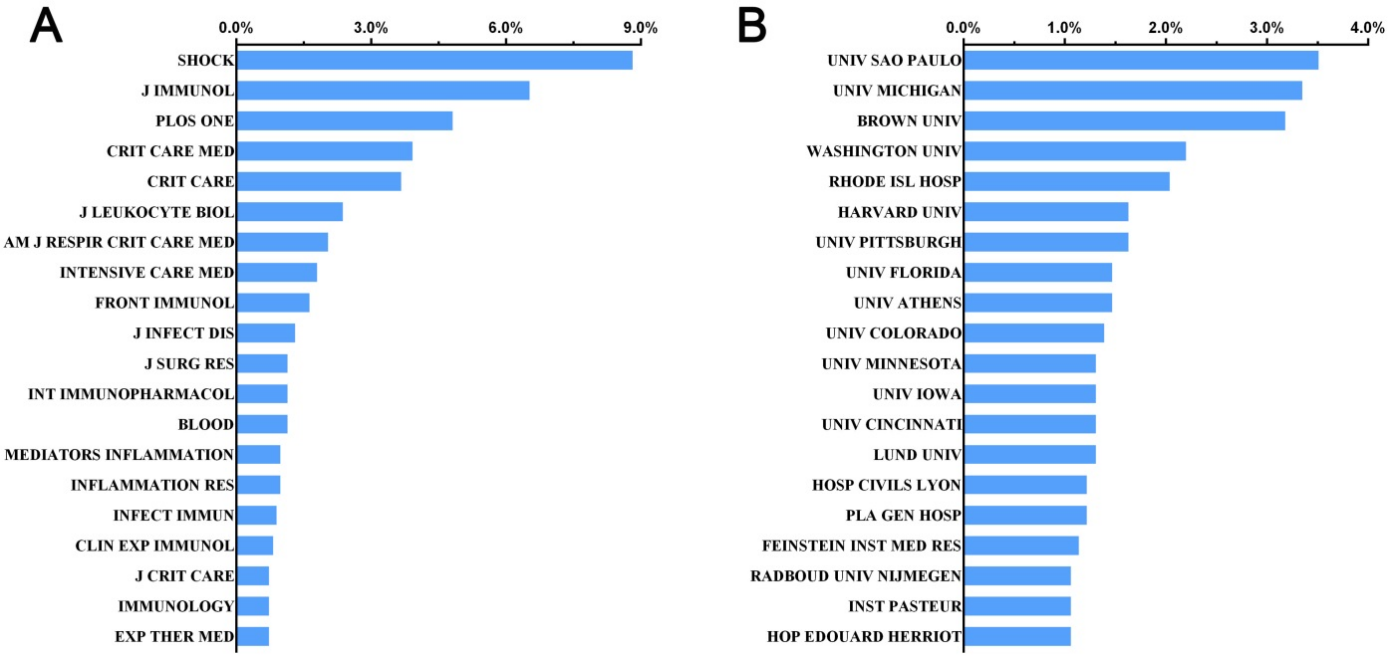
** Distribution of institutions and journals focusing on sepsis and immune response.** (A) Distribution of top 20 journals publishing research on sepsis and immune response; (B) Distribution of top 20 institutes undergoing sepsis and immune response.

Approximately half of the papers within this scope were published in the top 20 journals (565, 46.20%). The number of papers published on journal *Shock* (IF=3.005, 2017) was the highest with 108 records. Besides, *Journal of Immunology* (IF=4.54, 2017) ranked the second with 80 publications. *Critical Care Medicine* (IF=6.630, 2017) and* Critical Care* (IF=6.425, 2017) have published 48 papers and 45 papers, which ranked the fourth and the fifth, respectively. The journal *Intensive Care Medicine* (IF=15.008, 2017) ranked the eighth with a total of 22 publications in this field. Other journals with immense academic impact, the *Lancet* as an example, also published a high-quality clinical trial and a review in related field, as well as the *Journal of the American Medical Association (JAMA)* with a clinical trial. Additionally, we found six studies that were issued on journal *Nature Medicine*. The top 20 journals published the most papers were listed in the **Fig. 4A**.

### Institutions publishing researches on sepsis and immune response

The *University of Sao Paulo* in Brazil had the highest number of publications among institutions worldwide. Forty-three papers were documented by this affiliation, which accounted for 3.5% of all publications. Within the list of top 20 institutions in this field, there were 13 American institutions followed by 4 French institutions, 1 Brazil institution, 1 Swedish as well as 1 Greece institution. Of note, one Chinese institution the Chinese People's Liberation Army General Hospital was on the list and ranked the fifteenth with a total of 15 publications since 1999 (**Fig. 4B**).

### Authors publishing researches on sepsis and immune response

A total of 250 papers by the top 10 authors accounted for 20.4% of all literatures in related area. Ayala A from Rhode Island Hospital and Brown University had published 32 papers related to sepsis and immune response, which ranked the first in the number of publications. Both Cunha FQ and Monneret G had published 30 papers in total and ranked the second among all authors. As shown in **Table 1**, there were 6 authors from the United States, 3 from France, and 2 from Brazil. Notably, Hotchkiss RS from Washington University was the author with the highest citation frequency (3532 times in total), even though ranked the sixth with 22 publications (**Table 1 and Table 2**).

### Analysis of keywords in publications of sepsis and immune response

**Figure 5:**
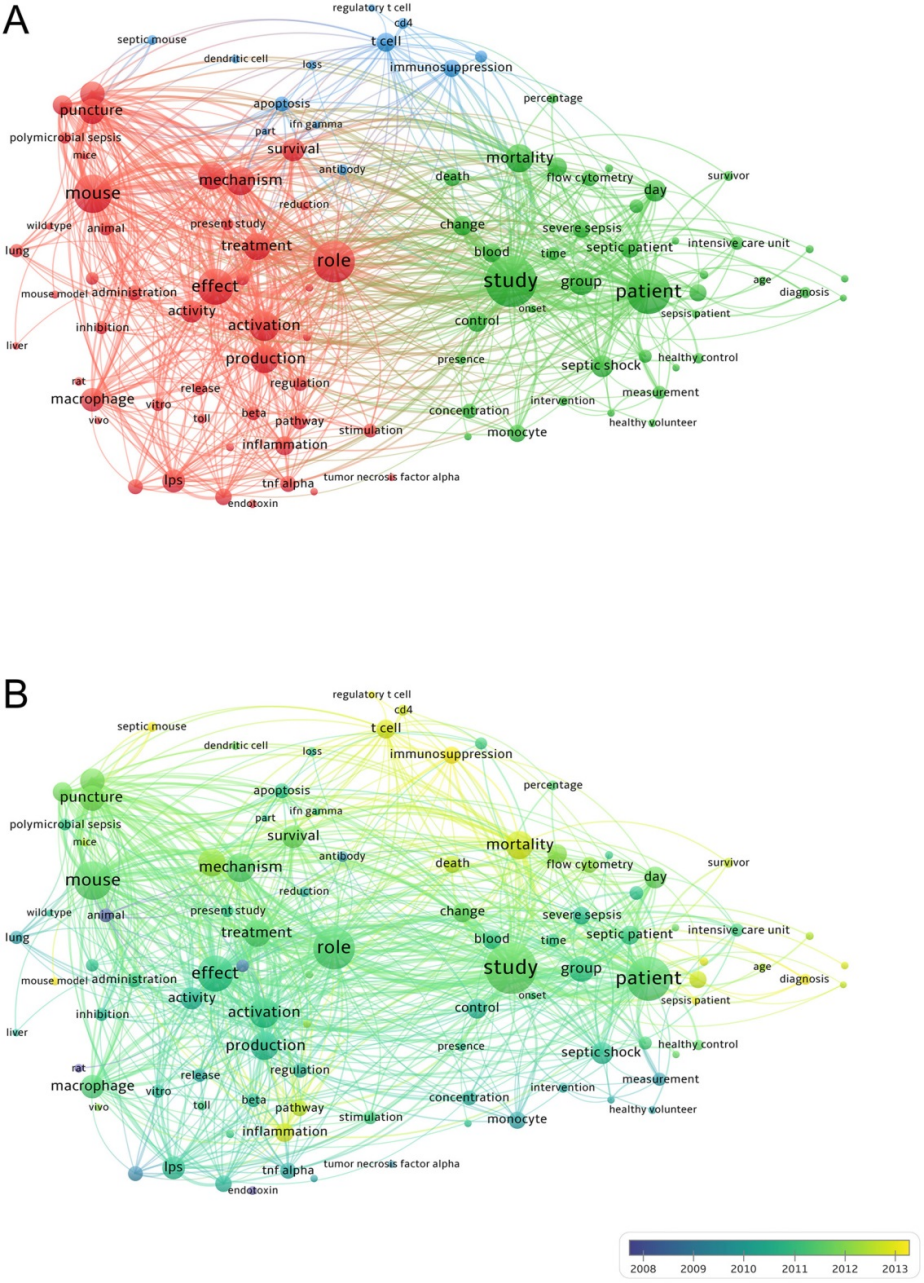
** The analysis of keywords in publications of sepsis and immune response.** (A) Mapping of the keywords in the area of sepsis and immune response. The words were divided into 3 cluster in accordance with different colors generated by default: inflammation related research (left in red), clinical research (right in green), and immunosuppression related research (up in blue). The circle with a large size represented the keywords that appeared at a high frequency; (B) Distribution of keywords was presented according to the appearance for the average time. The blue color represented for early appearance and yellow colored for keywords that appeared recently. Two keywords co-occurred if they both occurred on the same line in the corpus file. The smaller the distance between two keywords, the larger the number of co-occurrences of the keywords.

We analyzed keywords extracted from 1225 publications by applying VOSviewer. As shown in **Fig. 5A**, 100 keywords (defined as terms that occurred more than 50 times within titles and abstracts in all papers) were identified and classified into 3 clusters: “inflammation related research”, “clinical research”, “immunosuppression related research”. Within the cluster of “inflammation related research”, following keywords were frequently mentioned: mouse (435 times), effect (400 times), activation (319 times), mechanism (308 times), and production (299 times). As with the cluster of “clinical research”, relevant keywords were also listed, including study (624 times), patient (519 times), mortality (297 times), group (268 times), and septic shock (223 times). In cluster of “immunosuppression related research”, primary keywords within publications were T cell (185 times), immunosuppression (167 times), apoptosis (142 times), lymphocyte (109 times), as well as antibody (96 times). Detailed consequences with regard to the co-occurrence analysis of all incorporated keywords were presented in **Supplemental Table S1**.

As shown in **Fig. 5B**, VOSviewer colored all keywords in accordance with the average time when the word appeared. The blue color represented the word appeared relatively earlier upon time course, while keywords in yellow for recent appearance. During the early stage of the researches on sepsis and immune response, endotoxin (cluster 1, AAY of keywords was 2007.3) was the major topic in this field. More recently, research trends had implicated that “regulatory T cell” from the “immunosuppression related research” cluster might be a new target for studying sepsis and immune response (cluster 3, AAY was 2014.0). Within the first cluster (“inflammation related research”), the newest word was “mouse model” (cluster 1, AAY was 2013.4), which occurred for 65 times. In the second cluster (“clinical research”), “sepsis patient” (cluster 2, AAY was 2013.6) and “diagnosis” (cluster 2, AAY was 2013.3) were the recently emerging words, which appeared for 65 and 96 times, respectively. As for the third cluster (“immunosuppression related research”), “immunosuppression” (cluster 3, AAY was 2013.3) was noted as a new topic other than “regulatory T cell”, which appeared for 166 times.

## Discussion

### Research trends in sepsis and immune response

From the current study, it is clearly documented that the United States and Germany ranked the first and the second respectively in terms of total number of citations and value of H-index in the area of sepsis-associated immune dissonance. The United States has made the greatest contribution in researches on sepsis and immune response, which is evidenced by the number of publications, citation frequency, and H-index. Given the fact that the definition of sepsis-induced immunosuppression was initially proposed by American scholars, the United States focused on this issue earlier than that from the rest of the world. Additionally, the condition of basic medical researches and clinical trials appear to be superior in the United States, which showing advanced equipment, professional researchers as well as adequate funding. Aforementioned characteristics also indicate the United States occupying the leading position in this field.

Of note, China ranked the second for the total number of publications, while it ranked the eighth and the seventh for the citation frequency and H-index, respectively. The contradiction between the quantity and quality of publications in China might attribute to several reasons. Firstly, it was not until the year of 2005 did Chinese investigators publish articles in the related area, but remained relatively small in the number of publications before 2010. Thus, it takes more time for China to catch up with the citation frequency compared to other countries. Secondly, diagnosis of sepsis is far from standardization in China. In most hospitals of China, even in tertiary hospitals, personnel for medical care does not regularly assign Sepsis Related Organ Failure Assessment (SOFA) score to critically ill patients, which contributes to high omission rate for the diagnosis of sepsis. Thirdly, China presents with a lack of high-quality multicenter Randomized Clinical Trials (RCTs), but reports relatively more on observational studies, which may be insufficient for providing solid evidences in clinical practices.

As depicted in time curve, we observed a rapid growth of cumulative number of publications concerning sepsis and immune response globally since 2010. Although the number of publications per year has reached a stable range for many countries, including the United States and Germany, but the number of publications related to the subject sustains a rapid growth in China. Of note, the number of papers in recent 6 years surpasses the sum of publications in the past, and the number of publications comes from China accounted for a large proportion undoubtedly.

Although Germany, France, and Brazil had published fewer papers than that of China during 1999 to 2018, both their citation frequency and H-index surpassed those of China. Therefore, it reveals the urgent need in promoting the quality of papers for Chinese researchers in the future.

The United States owned 12 institutions from the top 20 institutions in researches with regard to sepsis and immune response, indicating its dominant role in this field. Institution (University of Sao Paulo) that had published the most articles in such area belonged to Brazil, while American institutions ranked from the second to the fifth. The United States possesses the most elite institutions around the world, which partially explains why the United States consistently maintains its leading position in research field regarding to sepsis-induced immunosuppression. Besides, there were 3 French institutions on the list as well. Only one institution (the Chinese People's Liberation Army General Hospital) was located in China. Thus, it requires more elite institutions from China to improve the international status in the important research direction associated with sepsis.

Notably, the journal of *Shock* had published 108 papers in the field, which was far ahead compared to others. Other journals including *Journal of Immunology*,* PLoS ONE*, and *Critical Care Medicine* were the primary journals involving in the publication of sepsis and immune response. Thus, it suggests that future development within this field might likely be presented in the aforementioned journals.

As for authors, Ayala A from the United States, Cunha FQ form Brazil, and Monneret G from France had published the most articles on sepsis and immune response. Ayala A mainly investigates the dysfunction of regulatory T cells and macrophages in the pathogenesis of sepsis 27-29, while Cunha FQ evaluates the potential role of neutrophils in sepsis and tries to attenuate septic lesions by regulating the function of neutrophils 30-32. Although Hotchkiss RS from Washington University merely ranked the sixth in the sum of publications, total citation frequency of his papers was the highest among the list. Hotchkiss RS is the pioneer of exploring precise mechanism underlying sepsis-induced immunosuppression, and his impressive reviews on sepsis and immune response have been cited for a great amount of times 7, 10. In addition, the cooperation between different authors makes great sense in studying sepsis and immune response. For example, Monneret G had been listed as a co-author in various manuscripts that belonged to aforementioned authors, indicating a close cooperation with different institutions as well as other authors. It is our belief that those researchers may play a unique yet indispensable role within the scope of sepsis and immune dysfunction, which would extensively affect the future development and predict the hotspot of this field simultaneously.

### Research focuses on sepsis and immune response

Published papers with the highest citation frequency are associated with tremendous academic impact on a certain area. The detailed information about the top 10 cited publications within sepsis and immune response was listed in **Table 2**. The paper entitled “Bone marrow stromal cells attenuate sepsis via prostaglandin E_2_-dependent reprogramming of host macrophages to increase their interleukin-10 production” had been cited for 1110 times since its publication, which was the most cited papers in related field. This study was published on *Nature Medicine* in 2009, whose corresponding author was Mezey E. They found that co-culture of macrophages with bone marrow stromal cells could induce release of interleukin-10 through prostaglandin E_2_ and prostaglandin EP2/EP4 receptors dependent manners, thereby it might serve as a potential therapeutic target in the treatment of sepsis 33. Both of the second and the third papers among the list belonged to Hotchkiss RS and his colleagues. These two articles, a clinical study and a review, were published in *JAMA* and *Nature Reviews Immunology*, respectively. Both publications strengthen the notion that sepsis-induced immunosuppression is a major abnormality among septic patients, which highlight remarkable clinical significance of immunotherapy for septic complications 7, 34. In fact, a total of 4 publications within the list of top 10 cited papers were reported by Hotchkiss RS, which also explained why he was the author with the highest citation frequency.

For the latest hotspot, “regulatory T cell” from cluster “immunosuppression related research” appears the most recently (cluster 3, AAY is 2014.0). Actually, there are 3 of 5 newly appeared words which come from “immunosuppression related research” cluster, including “septic mouse” and “immunosuppression”. As shown in** Fig. 5A** and **Fig. 5B**, the cluster of “immunosuppression related research” gained less attention when compared to the other two clusters. Nevertheless, this cluster contains several newly appeared words, suggesting that the mechanism of sepsis-induced immunosuppression is well accepted and extensively studied nowadays. In accordance with bibliometric map, the relationship between immunosuppression and T cells seems to be a promising hotspot for further researches, as disturbed response of T cell is noticed by sepsis environment in many dimensions. Other than apoptosis of CD4^+^ T cells, intrinsic defect of T cells is also characterized, as indicated by increased expression of several activation markers on circulating T cells following septic challenge, including glucose transporter type 1 (GLUT1), CD69, and signal transducer and activator of transcription 5 (STAT5) phosphorylation 35-37. Moreover, researchers have identified that co-inhibitory receptors such as PD-1, CTLA-4, T cell immunoglobulin mucin receptor 3 (TIM3), and lymphocyte activation gene 3 protein (LAG3) are all at high expression levels in both septic animal models and patients, which gave rise to the functional attrition of T cells 16, 17, 38. By targeting those co-inhibitory receptors, therapies for immune checkpoints appear to be capable of reversing immune dysfunction of T cells in the setting of sepsis 39-42.

Interestingly, metabolic reprogramming has been responsible for functional impairment of these cells, as shown by VOSviewer. Regulatory T cells (Treg) are reportedly involved in the pathophysiology of sepsis-induced immunosuppression as well, as noteworthy shift is evident from CD4^+^ T cells to Tregs under septic exposure, followed by compromised proliferation of T cells 28, 43, 44. The Tregs are noted with persistent activation and resistance to apoptosis during the course of sepsis, which constitutes a major threat for jeopardizing homeostasis of immune response 28. Downregulating the activity of Tregs shows great benefit for survival and outcome of septic animals through orchestrating balance between Th1/Th2 subtypes, indicating a noteworthy therapeutic target for sepsis 45. Recent study has been demonstrated that endogenous IL-33 plays an important role in promoting expansion of Tregs and inducing immune-paralysis in septic condition, which is further confirmed in IL-10 dependent manner 46. However, uncovering specific mechanism with regard to sepsis-induced functional changes of T cells would prompt us to make a breakthrough in finding novel drugs or therapies for clinical implications. In addition, animal studies with regard to dysregulated immune response in sepsis show weak connections to clinical investigations, hinting poor translational practice of pre-clinical sepsis researches. Multiple factors are responsible for this inadequate translation, involving unclarified standard for animal modeling, inappropriate study designs, as well as use of animal data without rigor. Standardization of preclinical sepsis studies from design to data interpretation would definitely improve translation of preclinical findings 47. Therefore, many attempts have been made in the cutting-edge research topics, which let us to assume that more publications with high-quality and clinical significance would raise our attention in the near future. In fact, multiple emerging issues and molecules are noted with critical involvement in sepsis and host immune response, covering inhibitory immune cells, neuro-endocrine immune networks, as well as negative immunomodulatory molecules, such as CTLA-4 and PD-L1. It is indeed essential for potential future developments in sepsis research, which urges for deep understanding into exploring novel mechanism and remedies for sepsis-induced immunosuppression, including dysfunction of both immune cells and immunomodulatory mechanisms, and combined control of both cellular fate and functional homeostasis of modulatory pathways.

### Strengths and limitations

Publications on sepsis and immune response assessed in the current study are extracted from the Web of Science database of Science Citation Index Expanded journals. The data analysis is relatively comprehensive and objective. Nonetheless, some limitations are inevitable. Due to our inclusion criteria, we enrolled publications only in English in the present investigation. Thus, important studies related to research area of sepsis and immune response in non-English language might be neglected and excluded from the database and analysis. In addition, papers published in 2019 were not incorporated in the current work, which means the analysis did not contain any keywords from 2019. All the search works were conducted within one day in order to avoid bias of updating database. However, we don't think that those latest publications possess considerable citation frequency, which might at least in part affect our conclusions. We believe that future works should address latest publications as well as citation frequency in non-English languages.

## Conclusions

Taken together, the current study has summarized the global research trends concerning sepsis and host immune response. The United States has made the biggest contribution within this important field. Although China has considerable quantity of publications, the quality of these papers literally needs further improvement. Latest studies and novel progress can be found in *Shock* and *Journal of Immunology*. Ayala A, Cunha FQ, Monneret G as well as Hotchkiss RS are all good candidates for academic collaboration in the area. Immunosuppression related researches have not been paid sufficient attention previously, while it has already turned into the hotspot topic recently, especially the precise mechanism and its modulatory strategy for immune dissonance in the development of clinical sepsis.

## Supplementary Material

Supplementary figures and tables.

## Figures and Tables

**Table 1:** Top 10 authors with most publications in research scope of sepsis and immune response

Author	Country	Affiliation	No. of Publications	No. of Citations
Ayala A.	USA	Rhode Island Hospital, Brown University	32	1943
Cunha FQ	Brazil	University of Sao Paulo	30	1637
Monneret G	France	Hospices Civils de Lyon, Edouard Herriot Hospital	30	2946
Chung CS	USA	Rhode Island Hospital, Brown University	29	1637
Venet F	France	Hospices Civils de Lyon, Edouard Herriot Hospital	25	1740
Hotchkiss RS	USA	Washington University School of Medicine	22	3532
Alves JC	Brazil	René Rachou Institute, Oswaldo Cruz Foundation	21	1240
Wang P	USA	The Feinstein Institute for Medical Research	16	308
Griffith TS	USA	University of Minnesota	15	256
Lepape A	France	Hospices Civils de Lyon, Edouard Herriot Hospital	15	1419
Moldawer LL	USA	University of Florida College of Medicine	15	1135

**Table 2:** Top 10 high-cited papers related to sepsis and immune response

Title	Corresponding authors	Journal	Publication Year	Total Citations
Bone marrow stromal cells attenuate sepsis via prostaglandin E_2_-dependent reprogramming of host macrophages to increase their interleukin-10 production	E´va Mezey	NATURE MEDICINE	2009	1110
Immunosuppression in patients who die of sepsis and multiple organ failure	Richard S. Hotchkiss	THE JOURNAL OF THE AMERICAN MEDICAL ASSOCIATION	2011	616
Sepsis-induced immunosuppression: from cellular dysfunctions to immunotherapy	Richard S. Hotchkiss	NATURE REVIEWS IMMUNOLOGY	2013	590
Protection from septic shock by neutralization of macrophage migration inhibitory factor	Calandra Thierry	NATURE MEDICINE	2000	561
Sepsis-induced apoptosis causes progressive profound depletion of B and CD4^+^ T lymphocytes in humans	Richard S. Hotchkiss	JOURNAL OF IMMUNOLOGY	2001	511
Nrf2 is a critical regulator of the innate immune response and survival during experimental sepsis	Shyam Biswal	JOURNAL OF CLINICAL INVESTIGATION	2006	505
Immunosuppression in sepsis: a novel understanding of the disorder and a new therapeutic approach	Richard S. Hotchkiss	LANCET INFECTIOUS DISEASES	2013	490
Pre-B cell colony-enhancing factor inhibits neutrophil apoptosis in experimental inflammation and clinical sepsis	John C. Marshall	JOURNAL OF CLINICAL INVESTIGATION	2004	442
MyD88-dependent expansion of an immature GR-1^+^ CD11b^+^ population induces T cell suppression and Th2 polarization in sepsis	Lyle L. Moldawer	JOURNAL OF EXPERIMENTAL MEDICINE	2007	395
Receptor for advanced glycation end products (RAGE) regulates sepsis but not the adaptive immune response	Peter P. Nawroth	JOURNAL OF CLINICAL INVESTIGATION	2004	362

## Abbreviations

WOS: Web of science
AAY: Average appearing years
ICU: Intensive care unit
DC: Dendritic cell
PD-1: Programmed death-1
CTLA-4: Cytotoxic T lymphocyte antigen-4
BTLA: B and T lymphocyte attenuator
SCI-E: Science citation index-expanded
RRI: Relative research interest
IF: Impact factor
JCR: Journal citation reports
JAMA: The Journal of the American Medical Association
SOFA: Sepsis related organ failure assessment
RCTs: Randomized clinical trials
GLUT1: Glucose transporter type 1
STAT5: Signal transducer and activator of transcription 5
TIM3: T cell immunoglobulin mucin receptor 3
LAG3: Lymphocyte activation gene 3 protein
Treg: Regulatory T cell
