# Supplementary material for: Publication Trends of Research on Sepsis and Host Immune Response during 1999-2019: A 20-year Bibliometric Analysis
Source: Int J Biol Sci. 2020 Jan 1;16(1):27–37. doi: 10.7150/ijbs.37496 (PMC6930382; doi:10.7150/ijbs.37496)
Supplement: Supplementary file 1 — Supplementary figures and tables. [file ijbsv16p0027s1.pdf]

**Supplemental Table 1. The analytic consequence of 100 keywords with at least 50 occurrence times**

| ID | Keywords | Cluster | Links | Occurrences | Average appearing years (AAY) | Average citations |
|---|---|---|---|---|---|---|
| 1 | activation | 1 | 99 | 319 | 2011.0 | 34.98 |
| 2 | activity | 1 | 99 | 225 | 2010.5 | 31.84 |
| 3 | administration | 1 | 98 | 137 | 2011.0 | 25.97 |
| 4 | admission | 2 | 82 | 67 | 2012.3 | 24.25 |
| 5 | age | 2 | 89 | 69 | 2012.2 | 35.55 |
| 6 | animal | 1 | 97 | 127 | 2008.1 | 35.00 |
| 7 | antibody | 3 | 99 | 96 | 2009.4 | 55.18 |
| 8 | apoptosis | 3 | 98 | 142 | 2010.9 | 35.95 |
| 9 | association | 2 | 95 | 74 | 2012.7 | 30.58 |
| 10 | beta | 1 | 99 | 126 | 2010.5 | 34.70 |
| 11 | blood | 2 | 99 | 184 | 2011.0 | 26.53 |
| 12 | c reactive protein | 2 | 80 | 56 | 2012.4 | 25.84 |
| 13 | cd4 | 3 | 98 | 95 | 2012.6 | 39.86 |
| 14 | cecal ligation | 1 | 96 | 254 | 2011.8 | 35.72 |
| 15 | change | 2 | 99 | 192 | 2011.6 | 25.05 |
| 16 | clp | 1 | 97 | 191 | 2011.7 | 28.77 |
| 17 | comparison | 2 | 98 | 64 | 2011.0 | 28.70 |
| 18 | concentration | 2 | 99 | 140 | 2010.2 | 35.71 |
| 19 | contrast | 1 | 99 | 111 | 2008.7 | 36.99 |
| 20 | control | 2 | 99 | 195 | 2010.4 | 30.76 |
| 21 | day | 2 | 99 | 219 | 2011.6 | 29.12 |
| 22 | death | 2 | 99 | 166 | 2012.4 | 49.04 |
| 23 | dendritic cell | 3 | 94 | 72 | 2011.6 | 30.42 |

| | | | | | | |
|---|---|---|---|---|---|---|
| 24 | diagnosis | 2 | 93 | 96 | 2013.3 | 17.65 |
| 25 | difference | 2 | 99 | 128 | 2010.7 | 34.01 |
| 26 | effect | 1 | 99 | 400 | 2010.9 | 36.45 |
| 27 | endotoxemia | 1 | 96 | 136 | 2009.2 | 35.22 |
| 28 | endotoxin | 1 | 95 | 73 | 2007.3 | 43.44 |
| 29 | enzyme | 2 | 99 | 60 | 2010.7 | 22.82 |
| 30 | flow cytometry | 2 | 99 | 149 | 2011.9 | 25.35 |
| 31 | group | 2 | 99 | 268 | 2011.1 | 29.43 |
| 32 | healthy control | 2 | 93 | 89 | 2011.4 | 27.00 |
| 33 | healthy volunteer | 2 | 90 | 66 | 2009.8 | 31.00 |
| 34 | ifn gamma | 3 | 96 | 66 | 2011.0 | 29.41 |
| 35 | ill patient | 2 | 98 | 61 | 2011.5 | 53.59 |
| 36 | immunosuppression | 3 | 99 | 167 | 2013.3 | 39.14 |
| 37 | induction | 1 | 97 | 116 | 2010.7 | 36.39 |
| 38 | inflammation | 1 | 99 | 185 | 2012.6 | 38.17 |
| 39 | inhibition | 1 | 98 | 121 | 2010.4 | 38.24 |
| 40 | innate immune response | 1 | 98 | 65 | 2011.0 | 54.82 |
| 41 | intensive care unit | 2 | 97 | 123 | 2011.2 | 46.50 |
| 42 | intervention | 2 | 99 | 82 | 2010.0 | 47.89 |
| 43 | lipopolysaccharide | 1 | 99 | 161 | 2010.4 | 38.40 |
| 44 | liver | 1 | 90 | 64 | 2010.4 | 27.34 |
| 45 | loss | 3 | 97 | 76 | 2010.6 | 54.43 |
| 46 | lps | 1 | 99 | 230 | 2010.7 | 34.97 |
| 47 | lung | 1 | 94 | 120 | 2009.8 | 47.39 |
| 48 | lymphocyte | 3 | 98 | 109 | 2011.2 | 29.33 |
| 49 | macrophage | 1 | 99 | 239 | 2011.3 | 33.39 |

| | | | | | | |
|---|---|---|---|---|---|---|
| 50 | main result | 2 | 97 | 61 | 2010.0 | 47.87 |
| 51 | marker | 2 | 99 | 165 | 2012.5 | 28.39 |
| 52 | measurement | 2 | 99 | 120 | 2009.5 | 45.40 |
| 53 | mechanism | 1 | 99 | 308 | 2011.4 | 40.49 |
| 54 | mice | 1 | 89 | 65 | 2012.2 | 21.71 |
| 55 | model | 1 | 99 | 291 | 2012.0 | 27.41 |
| 56 | monocyte | 2 | 99 | 169 | 2009.8 | 31.85 |
| 57 | mortality | 2 | 99 | 297 | 2012.6 | 35.20 |
| 58 | mouse | 1 | 97 | 435 | 2011.5 | 36.16 |
| 59 | mouse model | 1 | 92 | 65 | 2013.4 | 21.28 |
| 60 | onset | 2 | 99 | 73 | 2011.1 | 30.81 |
| 61 | outcome | 2 | 99 | 190 | 2011.9 | 29.21 |
| 62 | part | 3 | 97 | 57 | 2010.5 | 39.54 |
| 63 | pathway | 1 | 99 | 151 | 2012.4 | 32.24 |
| 64 | patient | 2 | 99 | 519 | 2011.4 | 33.61 |
| 65 | percentage | 2 | 99 | 87 | 2011.4 | 30.28 |
| 66 | polymicrobial sepsis | 1 | 92 | 106 | 2010.9 | 33.12 |
| 67 | presence | 2 | 99 | 84 | 2010.7 | 27.92 |
| 68 | present study | 1 | 99 | 111 | 2010.8 | 25.77 |
| 69 | production | 1 | 99 | 299 | 2010.7 | 37.32 |
| 70 | puncture | 1 | 97 | 266 | 2011.8 | 34.50 |
| 71 | rat | 1 | 92 | 70 | 2007.5 | 23.17 |
| 72 | reduction | 1 | 98 | 89 | 2010.2 | 47.02 |
| 73 | regulation | 1 | 99 | 142 | 2010.4 | 30.80 |
| 74 | regulatory t cell | 3 | 97 | 63 | 2014.0 | 22.78 |
| 75 | release | 1 | 97 | 119 | 2010.0 | 30.42 |

| | | | | | | |
|---|---|---|---|---|---|---|
| 76 | role | 1 | 99 | 481 | 2011.3 | 32.00 |
| 77 | sepsis patient | 2 | 95 | 65 | 2013.6 | 25.69 |
| 78 | septic mouse | 3 | 93 | 86 | 2013.4 | 23.91 |
| 79 | septic patient | 2 | 99 | 183 | 2011.0 | 30.98 |
| 80 | septic shock | 2 | 99 | 223 | 2010.7 | 40.76 |
| 81 | severe sepsis | 2 | 99 | 174 | 2010.6 | 40.25 |
| 82 | severity | 2 | 99 | 119 | 2011.2 | 27.38 |
| 83 | specificity | 2 | 80 | 61 | 2012.6 | 24.72 |
| 84 | stimulation | 1 | 99 | 124 | 2011.1 | 28.23 |
| 85 | study | 2 | 99 | 624 | 2011.5 | 30.94 |
| 86 | survival | 1 | 99 | 234 | 2011.6 | 33.82 |
| 87 | survivor | 2 | 96 | 82 | 2012.4 | 25.77 |
| 88 | systemic inflammation | 1 | 98 | 66 | 2012.0 | 26.09 |
| 89 | systemic inflammatory response | 1 | 98 | 65 | 2011.5 | 34.37 |
| 90 | systemic inflammatory response syndrome | 2 | 92 | 63 | 2011.5 | 26.03 |
| 91 | t cell | 3 | 99 | 185 | 2012.7 | 28.10 |
| 92 | time | 2 | 99 | 117 | 2010.9 | 38.15 |
| 93 | tnf | 1 | 96 | 62 | 2009.7 | 54.40 |
| 94 | tnf alpha | 1 | 99 | 152 | 2010.0 | 41.84 |
| 95 | toll | 1 | 97 | 82 | 2011.1 | 57.33 |
| 96 | treatment | 1 | 99 | 284 | 2011.4 | 34.91 |
| 97 | tumor necrosis factor alpha | 1 | 97 | 59 | 2009.3 | 37.92 |
| 98 | vitro | 1 | 98 | 119 | 2010.5 | 33.95 |
| 99 | vivo | 1 | 93 | 60 | 2012.0 | 35.20 |
| 100 | wild type | 1 | 89 | 58 | 2010.5 | 37.50 |
